# Spectroscopic Determination of Aboveground Biomass in Grasslands Using Spectral Transformations, Support Vector Machine and Partial Least Squares Regression

**DOI:** 10.3390/s130810027

**Published:** 2013-08-06

**Authors:** Miguel Marabel, Flor Alvarez-Taboada

**Affiliations:** GEOINCA-202, University of León, Campus of Ponferrada C/ Avda, de Astorga, 24401 Ponferrada, León, Spain; E-Mail: mmarag00@estudiantes.unileon.es

**Keywords:** biomass, continuum removal, spectrometer, hyperspectral, radiometry, Area Over the Minimum, Maximum Band Depth, PLSR, SVM, OLSR

## Abstract

Aboveground biomass (AGB) is one of the strategic biophysical variables of interest in vegetation studies. The main objective of this study was to evaluate the Support Vector Machine (SVM) and Partial Least Squares Regression (PLSR) for estimating the AGB of grasslands from field spectrometer data and to find out which data pre-processing approach was the most suitable. The most accurate model to predict the total AGB involved PLSR and the Maximum Band Depth index derived from the continuum removed reflectance in the absorption features between 916–1,120 nm and 1,079–1,297 nm (R^2^ = 0.939, RMSE = 7.120 g/m^2^). Regarding the green fraction of the AGB, the Area Over the Minimum index derived from the continuum removed spectra provided the most accurate model overall (R^2^ = 0.939, RMSE = 3.172 g/m^2^). Identifying the appropriate absorption features was proved to be crucial to improve the performance of PLSR to estimate the total and green aboveground biomass, by using the indices derived from those spectral regions. Ordinary Least Square Regression could be used as a surrogate for the PLSR approach with the Area Over the Minimum index as the independent variable, although the resulting model would not be as accurate.

## 1. Introduction

Biomass is one of the strategic biophysical variables of interest in vegetation studies, regardless of being in cultivated or natural areas [1]. Aboveground biomass (AGB) can be defined in terms of fresh matter weight or dry matter weight, these two variables being strongly related, as well as water content [2]. The possibility of estimating the vegetation biomass and its modelling can aid in crop and bioenergy management [3], regarding the estimation of the yield and the management of its residuals [4]. It is also crucial due to its direct relationship with carbon and the holistic study of these systems as carbon sinks [5].

Measuring biomass directly is a destructive and expensive procedure [6], so researchers and managers are looking for non-destructive and repeatable methods to monitor biomass [7]. Remote sensing techniques meet the two previous requirements, and in addition, they allow both spatial and temporal analyses [8]. Some of the studies conducted in the past were related to data characterised by a high spectral resolution in the electromagnetic region between 400–2,500 nm, as a result of the absorption features in the reflectance curves [9–13]. Simple approaches using vegetation indices derived from the red and the near infrared (NIR) bands (e.g., simple ratio, normalised vegetation index) have been widely used to estimate biomass (e.g., [14,15]). Nevertheless, several studies have showed that the computation of narrow banded indices from broad bands can be inadequate to estimate biomass, due to variations in the colour of the soil, the canopy structure and/or atmospheric conditions [15]. Moreover, the NDVI that is calculated using these data can reach an asymptotic value once a certain biomass value is reached [16]. In contrast, some studies have found that indices computed from specific narrow-bands (hyperspectral data) improve biomass estimation [17,18]. In this context, the application of spectral transformations and statistical techniques that consider continuous regions of the spectrum is outlined as an opportunity to improve the models to estimate aboveground biomass [7].

Hyperspectral measurements of vegetation canopies obtained from hand-held spectroradiometers [7,10,12,13] or airborne sensors [15,19,20] contain useful information for the characterisation of vegetation, which could not be retrieved from multi-spectral imagery previously. However, these data sets contain large amounts of redundant information [21,22]. Also they are more affected by a lower signal-to-noise ratio. These two shortcomings have not deterred researchers from using hyperspectral datasets to model biophysical variables, but they have encouraged the development of techniques to overcome them.

The strong multicollinearity caused by a number of samples much smaller than the number of spectral bands considered as independent variables results in high correlation among the predictors and unreliable models [23]. One well known approach that can be used to avoid this problem is the selection of a statistical technique which can take into account multicollinearity [24]. Two of the most dominant approaches in this area are listed in Table 1 (Partial least square Regression—PLSR—and Support Vector Machine—SVM-), showing as well some valuable studies related with the estimation of vegetation biophysical variables. PLSR and SVM are full spectrum methods which have been widely used in chemometrics [25] and lately in studies related to the estimation of biomass from hyperspectral data [7,20,26]. Ordinary Least Squares Regression (OLSR) has been successfully used in some of these studies, albeit it required a previous selection of the input data (*i.e.*, only a limited number of independent features) [18,27].

In order to improve the signal-to-noise ratio of these data and enhance the information related to the biophysical variables, different pre-processing transformations have been applied to transform spectral data, preparing them for modelling. Pre-processing transformations of spectral data have been proved to improve the accuracy of prediction models [15,20,32,41–43]. Some of the most common transformations include: smoothing, averaging, normalisation, scatter correction, baseline correction, and derivatives [32], while the most widely used regarding AGB estimation is the continuum removal (CR) transformation [15,18,20,44]. In addition, the use of indices derived from the continuum removed spectrum has yielded accurate models to predict AGB and related biophysical variables [18,27]. Although some pre-processing transformations have been proposed, the choice of which pre-processing transformation to use might be related to the statistical technique and the region of the spectra considered as input data.

The main objective of this study was to evaluate the performance of two advanced statistical techniques (PLSR and SVM) for estimating the aboveground biomass from field spectrometer data and to find out which data pre-processing approach was the most suitable. The total dry aboveground biomass (TAGB) was considered as the target variable, as well as the green fraction of the dry aboveground biomass (as an absolute value (GAGB) and as a percentage of the total dry aboveground biomass (%GAGB)). In addition, several data pre-processing techniques were tested in order to reduce the noise in the data and to boost the accuracy of the statistical methods. Thus, the following approaches were compared: (i) PLSR applied to different parts of the spectrum (not transformed and transformed by the continuum removal and other transformation methods), (ii) PLSR applied to indices derived from the continuum removal transformation, (iii) SVM regression applied to different parts of the spectrum, and (iv) OLSR applied to indices derived from the continuum removal transformation (as a reference).

## 2. Material

### 2.1. Study Area

This study was developed in two adjacent grassy areas located in the municipality of Villanueva de La Cañada (Madrid, Spain) and is defined by their central coordinates ETRS89 UTM30 4163814478513 and ETRS89 UTM30 4164634478505 (in metres). Both test areas were covered by commercial grass/clover (*Lolium perenne*, *Poa pratense* and *Trifolium repens*) and were irrigated and coetaneous. 30 sample plots were placed in the study area in order to estimate their biomass and to be characterised radiometrically. Each 1 m × 1 m plot was established in a 2 m × 2 m homogeneous part of the grassy area. Each plot was then divided into four subplots (50 cm × 50 cm), which were the smallest sample units considered in this research. In these subplots, aboveground biomass was collected and spectral data were recorded. The field work was conducted on the 22 July 2012 and the plot locations were determined using a GNSS Topcon Hiper II. The GPS data was post-processed using reference stations in order to refer the coordinates to ETRS 89.

### 2.2. Canopy Reflectance Measurements

For each 50 cm × 50 cm subplot the top of the canopy reflectance was measured. Spectral data was gathered in a spectral range of 350–2,500 nm using an ASD FieldSpec^®^4 spectroradiometer. Hand held measurements were made with a 1.5 m fiber optic (25° field of view) from a height of about 1.5 m above the ground under clear sky conditions and around solar noon. Spectral readings were recorded in 1 nm intervals with a spectral resolution of 3 nm in the visible and near infrared spectra (VNIR detector: 350–1,000 nm) and 8 nm in the near and shortwave infrared (SWIR1 detector: 1,000–1,800 nm and SWIR2 detector: 1,800–2,500 nm). For each subplot, 15 reflectance readings were recorded, each one representing the average of 25 individual measurements of 100 ms, which increases the signal-to-noise ratio of the resulting measurement [7]. Before taking the spectral readings in each subplot, the spectroradiometer was calibrated against a reference panel of known reflectivity (Labsphere Spectralon^®^) in order to be able to convert the readings into absolute reflectance. The reflectance measurements attained for each subplot were used to characterise each 1 m × 1 m plot.

### 2.3. Dry Aboveground Biomass Measurements

**Figure 1.**
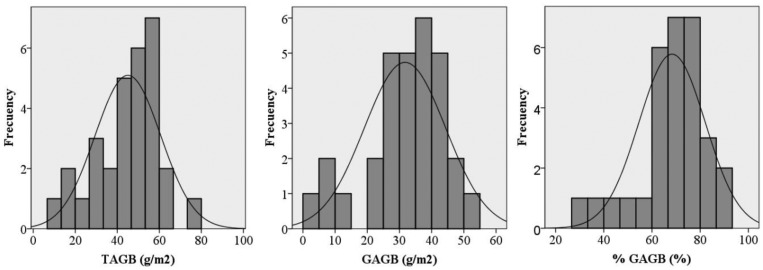
Distribution of frequencies for TAGB (total aboveground biomass), GAGB (green portion of the AGB) and % GAGB (Percentage of the green faction of the AGB).

All of the aboveground biomass in each 50 × 50 cm subplot located in the NE corner of each plot was harvested right after the spectral measurements were taken. In order to avoid a loss of water in the samples, they were put individually into hermetic plastics bags and immediately taken to the laboratory in portable fridges. The samples were weighed in the laboratory using a digital precision scale, therefore obtaining the total biomass weight. Afterwards, each sample was split in dry material and green material, in order to distinguish the green and dry fraction of the aboveground biomass. The green and dry fractions of each sample were separately dried in the oven for 48 h at 65 °C. After drying, samples were weighed again to determine the dry matter weight for both fractions. This workflow allowed the total dry aboveground biomass weight (TAGB) to be obtained, as well as the green fraction of the dry aboveground biomass weight (as an absolute value (GAGB) and as a percentage of the total dry aboveground biomass (%GAGB)). The total dry aboveground biomass weight (TAGB) was used as surrogate for the aboveground dry biomass (AGB) in each plot [35]. The biomass was determined by dividing the weight of the harvested grass by the surface area of the plots (expressed as g/m^2^). Table 2 summarises the descriptive statistics for the 30 subplots of the grass/clover, while Figure 1 depicts the distribution of frequencies of the sample for the three variables. The Shapiro-Wilk test for normality showed that the three variables were normal (α = 0.01).

## 3. Methods

### 3.1. Workflow

**Figure 2.**
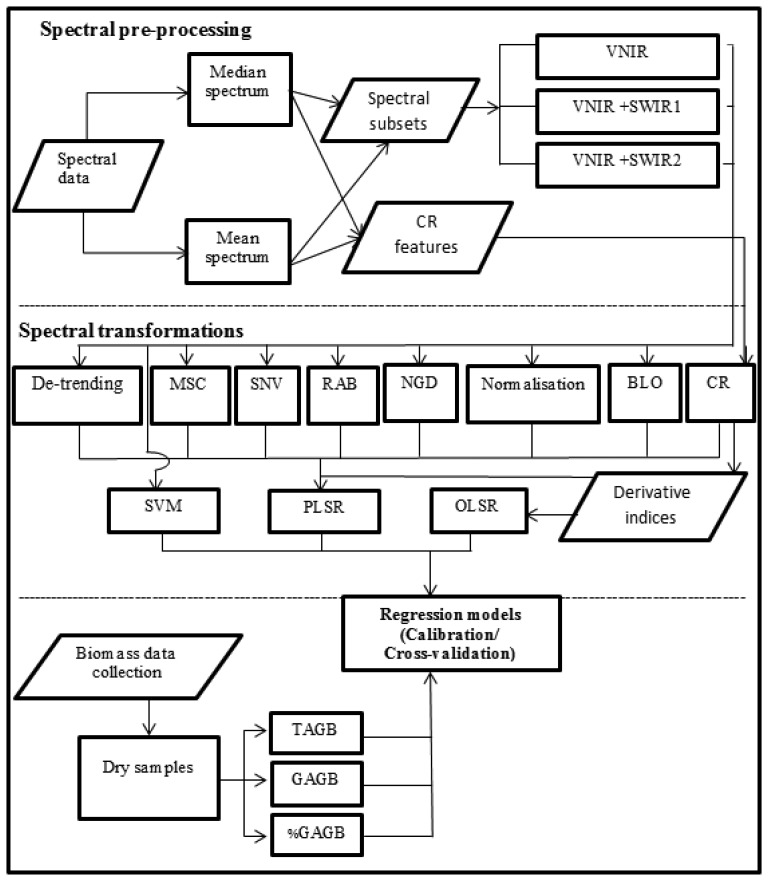
Methodology flowchart.

The methodology involved two main steps: spectral data processing and statistical analysis (Figure 2). The spectral data processing consisted of pre-processing the spectral data and applying different transformations to the spectra. Moreover, some indices were derived from the transformed data. Afterwards, spectral data was modelled to estimate TAGB, GAGB and %GAGB using Support Vector Machine, Partial Least Squares and Ordinary Least Square regressions. The following sections describe the processes depicted by Figure 2.

### 3.2. Spectral Data Processing

#### 3.2.1. Pre-Processing

The spectral data (absolute surface reflectance) was pre-processed to diminish the sensor noise. This step consisted of two tasks: averaging the 15 spectra measured for each subplot and identifying the noisiest wavelengths. Firstly, the radiometry of each subplot was characterised by the median and the mean spectrum of the 15 original measurements and averaged for the 1 × 1 m plot. Secondly, the wavelengths were grouped into three spectral subsets, taking into account the three different sensors which define the spectroradiometer (VNIR, SWIR 1, SWIR 2). The wavelengths from 1,360 nm to 1,385 nm, from 1,800 nm to 1,930 nm and above 2,400 nm were eliminated due to high amounts of noise [7]. Table 3 shows the wavelengths included in the three spectral subsets considered in this research: (i) VNIR; (ii) VNIR + SWIR 1; and (iii) VNIR + SWIR 1 + SWIR 2.

#### 3.2.2. Spectral Filtering and Transformations

In this comparative study, two groups of spectral transformation methods were applied: derivatives/transformations and continuum removal. This section covers the different types of preprocessing transformations which have been widely used by researchers to preprocess hyperspectral data (Table 4). In this study, a total of 19 pre-processing transformations, which prepared the biomass spectral curves (mean and median spectra) for multivariate calibration, were compared. These included: Norris derivatives [45], baseline offset, standardisation, reflectance to absorbance transformation, multiplicative scatter correction, normalisations and standard normal variate transformation [46]. Table 4 shows the complete list of pre-processing transformations tested, with their respective optional parameters. Hence, the transformations of the reflectances were used in the analysis rather than the reflectances themselves, in order to eliminate sensor noise and improve the performance of the model to estimate AGB. The analyses were carried out using the Unscrambler^®^ X 10.2 software (CAMO Software Inc., Woodbridge, Norway).

The Standard Normal Variate (SNV) is applied to spectroscopy data to remove the scattering effects, and it minimises the multiplicative interferences of the scattering caused by particles of different sizes [53]. In this transformation each spectrum is transformed individually by removing the intensity offset and scaling to unity standard deviation [51,52] and it has been widely used in many applications due to the normalisation of the spectra (*vid*. Table 4). Another method which attempts to reduce the scatter effects is the Multiplicative Scatter Correction (MSC). The MSC is based on adjusting all observations to an ‘ideal’ spectrum, so that the mean spectrum of all observations is used as reference and all spectra are affine estimated relative to this reference [50]. It should be noted that the MSC is therefore sensitive to the mean spectrum and it has to be recomputed any time new observations are added to the dataset. The characteristics of each MSC method applied to the spectra are described in [46].

Another option to model and correct the background interference is the De-trending method, especially when a constant, linear, or curved offset is present [51]. This method fits a polynomial of a given order (in this study: 1st, 2nd and 3rd order) to the entire sample and subtracts this polynomial from the spectrum, eliminating general or common components in the spectra [19]. In contrast to the baseline method, the De-trending method fits the polynomial to all points, baseline and signal. The baseline correction (defined by an offset) was also tested to eliminate the background noise from the data.

The transformations involving derivatives allow increasing differences among the overlapping and wide bands of the spectra, correcting as well the baseline effects [46]. The first derivative eliminates the baseline displacements which are parallel to the horizontal axis. The method that was applied in this study was the Norris gap first derivative [45]. The Savitzky-Golay method, which includes a simultaneous smoothing of the spectra, was tested as well, but the results were not as promising as the ones obtained by the other methods, so it was not included in the final set of transformations.

On the other hand, normalisation methods try to correct the effect of multiplicative factors on the original values of a variable. These methods identify a characteristic in a sample which should remain constant regardless of the considered sample and correct the scale of all the variables using that characteristic. In this study, the variables were normalised by the maximum value, the mean, the range, the area and the unit vector [46].

#### 3.2.3. Continuum Removal Transformation and Derived Indices

**Figure 3.**
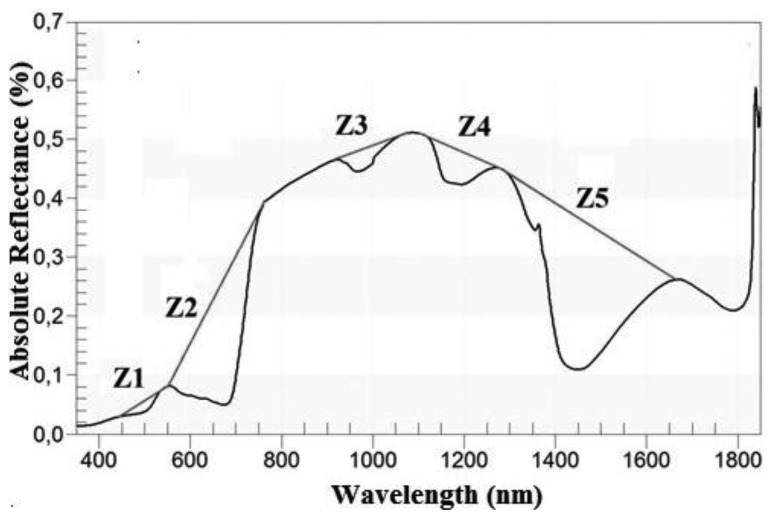
A grass reflectance spectrum and the representation of its continuum and absorption features (Zi: Zone I, as defined in Table 5).

In addition to the transformations described in the previous section, the Continuum removal transformation (CR) of the spectra was tested (Table 4). This technique is used to minimise the noise effects and to enhance the absorption characteristics of the spectrum [13]. The CR transformation is obtained by dividing the original reflectance values by the corresponding values in the continuum (*i.e.*, the segment which represents the trend) [39]. In order to apply this method, it was necessary to identify the limits of the regions where it was going to be performed. These regions were determined empirically [13] by taking account of the locations of the local spectral maxima of the grass, as long as those areas were sensitive to changes in the variable of interest (in this case, AGB). Hence, five zones (Z*i*) were identified and defined by their wavelengths (Table 5). Each zone corresponded with an absorption feature, as showed by Figure 3. Zones in the Z1 and Z2 domain have been successfully used in previous works to estimate leaf biochemistry [13,23], as well as for the classification of gramineae [48]. Likewise, absorption features located in Z3, Z4 and Z5 have been effective to model the variation in leaf water content [1,49,54,55]. For the present work, the limits of each zone had to be redefined to adjust them to the sample. The possibility of applying CR in the region between 1,800–2,100 nm was rejected, due to the low signal-to-noise ratio [44].

In addition to the continuous spectra derived from the CR transformation for each zone (continuum removed reflectance (CRR)), the absorption features were characterised by two indices: the maximum band depth (MBD) and the area over the minimum (AOM) [19]. The band depth (MBD) is the magnitude of the maximum difference between the spectrum and the continuum [39] and it is related with the intensity of the absorption in that region [1]. The area over the minimum (AOM) is described as the product between the depth and the width (*i.e.*, width measured at half of the depth) [27]. Both indices have succeeded in the estimation of biomass [1] and water and leaf biochemicals [18,21,27]. Both indices were computed using IDL.

### 3.3. Statistical Methods

This study tested three statistical methods for developing models to estimate biomass from the grass/clover spectra: partial least squares regression (PLSR), support vector machine (SVM) and ordinary least squares regression (OLSR). Due to its simplicity, the latter was included in the analysis as a reference and as a baseline to compare the results achieved by using PLSR and SVM.

#### 3.3.1. Partial Least Squares Regression (PLSR)

PLSR is a generalisation of linear multiple regression which is able to reduce the large number of measured collinear spectral variables to a few noncorrelated latent variables or factors [25,28]. Thus, this method builds a linear model based on the latent variables of the mean-centred matrix containing the predictor variables (the spectral bands in this study). In this regard, PLSR is closely related to principal component regression. The main difference is that, principal component regression decomposes first the spectra into a set of eigenvectors and scores and then regresses them against the response variables as a separate step, while PLSR uses the response variable information during the decomposition process [15]. One of the advantages of PLSR in spectroscopy is that it allows working with continuous parts of the spectra, handling collinear data and considering all the available wavelengths [7]. A comprehensive description of the PLSR algorithm can be found in [25].

As independent variables, the following data sets were considered: (i) pre-processed but not transformed data (spectral subsets defined in Table 3: VNIR, VNIR + SWIR1, VNIR + SWIR1 + SWIR2); (ii) spectral subsets VNIR, VNIR + SWIR1, VNIR + SWIR1 + SWIR2 after spectral filtering and transformations (Table 4); (iii) continuum removed reflectance (CRR) for Z1-Z5 (Table 5); (iv) derived indices from the continuum removed reflectance: maximum band depth (MBD) for Z1-Z5 (Table 5); and (v) derived indices from the continuum removed reflectance: area over the minimum (AOM) for Z1-Z5 (Table 5). An independent PLSR was fitted for the corresponding subsets in each dataset, since it has been showed that an accurate selection of the input data leads to a better performance of the method [15]. In addition, and with a comparative purpose, PLSR was applied to the full spectra (pre-processed but not transformed, and after excluding the noisy regions).

The selection of the most suitable model for each variable took into consideration the strategies to build a solid model [31]: small number of latent factors, small error in the prediction of the cross-validation, small adjusted error in the cross-validation and a coefficient of determination (R^2^) as close to 1 as possible. The optimal number of PLSR factors or latent variables to include in the model was selected by using the leave-one-out cross-validation method [1,7,19,25]. In order to maintain model parsimony, the criterion to add an additional factor to the model was that it had to reduce the root mean square error of cross-validation (RMSE) by >2% [1,15,33]. The RMSE was determined from the residuals of each cross-validation phase. Moreover, it was checked that the differences in the variance explained by the adjusted model in the calibration and cross-validation stages were not large. The performance of PLSR models was compared using the number of factors, and RMSE (absolute and percentage of the mean/median value of the variable) and the coefficient of determination (R^2^) for the cross-validation. The analyses were carried out using the Unscrambler^®^ X 10.2 software (CAMO Software Inc., Woodbridge, Norway).

#### 3.3.2. Support Vector Machine (SVM)

Lately, the use of support vector machines (SVMs) on various classification and regression problems has become increasingly popular and it has been successfully used in the estimation of grassland biomass [35], leaf area index [37] or leaf biochemical variables [20,36] using remotely sensed data.

Initially, SVM was developed to solve classification problems but it was later extended to also handle regression [56]. In regression, the goal is to estimate an unknown continuous-valued function based on a finite number set of noisy samples. Support vector regression (SVR) uses the principle of structural risk minimisation to simultaneously optimise performance and generalisation, and is often able to find non-linear and unique solutions [20]. There are a few different variants of SVR that utilise different optimisation algorithms, and the two that are commonly used are ε-SVR and η-SVR. The ε-SVR transforms the input data into a high-dimensional feature space using a non-linear function, solving the final model in a way that not only the training error is minimised, but also the complexity of the model [20]. A comprehensive description of both methods can be found in [57].

The ε-SVR method was applied in this study to estimate aboveground biomass, using the Vapnik's ε-insensitive loss function to minimise the training errors, which were not penalised as long as they were smaller than ε. As part of the process, a kernel function was applied, in order to map the data into a new space followed by finding the support vectors for the best performance for the type of model. The kernel type considered in this study was the linear kernel, since it is the one which requires the least parameters to be defined and because it is not as susceptible to overfitting as the radial or polynomial kernels [20]. The quality of the SVM models depends on a proper setting of the SVM meta-parameters: parameter ε and the parameter C [58]. The first one controlled the width of the epsilon-insensitive zone, used to fit the training data, and its value can affect the number of support vectors used to construct the regression function. Thus, the bigger the epsilon, the fewer support vectors selected [56], while bigger ε values result in more ‘flat’ estimates [37]. The parameter C determined the balance between the model complexity and the degree to which deviations larger than epsilon are tolerated in the optimisation. Therefore, larger values of C aim to minimise the empirical risk regardless of the complexity of the model.

A general methodology consisting of the following steps was applied [59]: (1) a simple scaling was applied to the training data (in order to avoid the over-weighting due to the features presenting the highest absolute values); (2) then, the lineal kernel was selected and the determination of parameters C and ε was solved by cross validation and grid search on the training data set, keeping the value of ε equal to 0.1 and γ as 1. Finally, (3) the estimated parameters were applied to the dataset used for cross-validation (previously scaled), and the accuracy statistics were computed.

In order to find the simplest model with an acceptable error and to maintain model parsimony, the criterion to add an additional support vector to the model was that it had to reduce the root mean square error of cross-validation (RMSE) by at least 2%. The RMSE was determined from the residuals of each cross-validation phase. In order to avoid an overfitting, it was checked that the RMSE values from the calibration and cross-validation stages were as well smaller than 2%. The performance of the SVM models was compared using the number of support vectors, the RMSE (absolute and percentage of the mean/median value of the variable) and the coefficient of determination (R^2^) for the cross-validation. The analyses were carried out using the Unscrambler^®^ X 10.2 software (CAMO Software Inc.).

#### 3.3.3. Ordinary Least Squares Regression (OLSR)

Ordinary Least Squares Regression (OLSR) was carried out using the biomass measurements (TAGB, GAGB, %GAGB) as dependent variables, and as independent variables the derived indices from the continuum removed reflectance (i) maximum band depth (MBD) for Z1–Z5 (Table 5) and (ii) area over the minimum (AOM) for Z1–Z5 (Table 5). These two sets of variables were chosen as input data due to their positive results in similar studies [1,13,19,27]. Continuous regions of the reflectance spectrum were rejected as input data for this method, since it tends to overfit the model and sometimes the selection of bands fails to correspond with known absorption bands [19]. The validation of the models was similar to the one described for PLSR, by means of a leave-one-out cross-validation method [1,7,19,25] and using as comparative criteria the RMSE (absolute value and percentage of the mean/median value of the variable) and the coefficient of determination (R^2^) of the cross-validation. The analyses were carried out using the Unscrambler^®^ X 10.2 software (CAMO Software Inc).

#### 3.3.4. Cross-validation Statistical Indicators

Overall, the results of the statistical models tested in this study were assessed in terms of coefficient of determination of the cross-validation (R^2^), the RMSE of the cross-validation (absolute value and percentage of the mean/median value of the variable) and the agreement between wavelengths/region identified as important by statistical analysis and known water/biomass absorption features [19]. In order to consider one model more accurate than another one, the former had to reduce the root mean square error of cross-validation (RMSE) by at least 2% [1]. A complete description of the cross-validation procedure and its aptitude to detect outliers and its capability of providing nearly unbiased estimations of the prediction error can be reviewed in [21,30,60].

## 4. Results and Discussion

On the whole, 140 models were tested for each of the three dependent variables (total, green and percentage of green grass/clover biomass), 12 of them without transformations of the spectral data and using PLSR and SVM, 124 involving PLSR and transformations/indices and four considering indices from the continuum removed spectra and OLSR. Thus, 420 models were explored in order to find suitable combinations among the regression method, the transformation type, the spectral subset/zone/index and the averaging method of the spectra for the estimation of biomass in grasslands. The results of these approaches are presented in the next section and discussed later on.

### 4.1 Results for the Estimation of above Ground Biomass

As a result of the comprehensive analysis of the relationships between total, green and percentage of green grass/clover biomass and the spectral data (transformed and non-transformed), Table 6 shows the best results achieved by the approaches which were tested. Due to the large amount of results obtained, only the following output is on display: results of using PLSR and SVM on each spectral subset (not transformed), results of applying the most accurate method to each spectral subset, results of the most accurate approach combining PLSR and CRR, and also PLSR and the indices derived from CCR, and finally, the results corresponding to the most accurate method involving OLSR. The results are showed in decreasing order of accuracy (R^2^) for each variable. The results depicted in Table 6 are commented on in Sections 4.1.1., 4.1.2., and 4.1.3, for each independent variable.

Transformations: *vid*. Table 4; CRR, MBD, AOM are the continuum removed reflectance and indices; Input data: *vid*. Table 3 and Table 5; Spectra is the average measurement of the data, F/C is the number of latent factors (PLSR) or parameter C (SVM); R^2^ is the coefficient of determination (cross-validation); RMSE is the Root Mean Square Error (cross-validation); %RMSE is the percentage of Root Mean Square Error (cross-validation) in relation to the average value of the variable.

#### 4.1.1. Results for the Estimation of Total above Ground Biomass

As shown in Table 6, PLSR models produced lower ranges of RMSE than SVM or OLSR when the same input data were considered. The most accurate model to predict TAGB involved PLSR and the MBD index derived from the continuum removed reflectance in the absorption feature between 916 and 1120 nm (Z3) and 1079 and 1297 nm (Z4) (RMSE = 7.120 g/m^2^, 15.81% of the mean value). The combination of PLSR and continuum removed spectra produced lower ranges of error for the cross validation analyses (RMSE = 7.120 to 7.136 g/m^2^) compared to the PLSR and the spectra transformed by other techniques (Normalization or Multiplicative Scatter Correction) (RMSE = 7.443 to 7.640 g/m^2^) (Table 6). However, transformations yielded more accurate PLSR models than non-transformed data.

**Figure 4.**
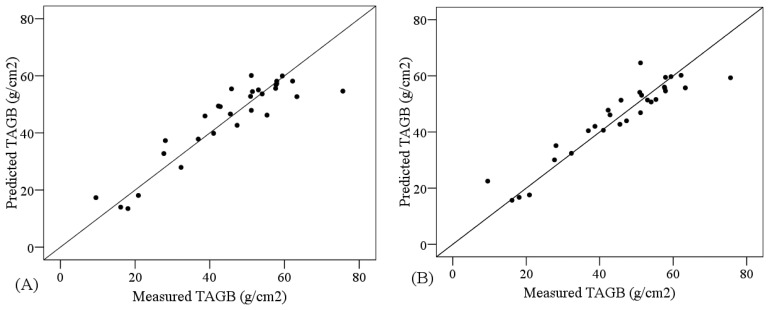
Cross-calibration results for TAGB using (**A**) PLSR based on the continuum-removed MBD index and (**B**) SVM based on non-transformed VNIR + SWIR1 data. One-to-one line is showed.

The comparative analysis of the performance of PLSR models and SVM models showed higher R^2^ and smaller RMSE for the PLSR models, regardless of the non-transformed spectral subset used as input data. In that case, the most accurate models were obtained using the VNIR+SW1 subset, for both PLSR (R^2^ = 0.756, RMSE = 7.866 g/m^2^) and SVM (R^2^ = 0.751, RMSE = 7.684 g/m^2^) (Table 6). In order to compare the PLSR and SVM approaches Figure 4 shows the suitability of the most accurate PLSR and SVM models, depicting the cross-validation results. Both models were suitable since the measured and predicted values are distributed along the one-to-one line and close to it.

OLSR provided the best results when using the AOM index derived from the continuum removed reflectance in the absorption feature between 1,079 and 1,297 nm (Z4) (RMSE = 8.150 g/m^2^, 18.09% of the mean value) (Table 6). This approach turned out to be more accurate and simpler than using PLSR or SVM and the non-transformed reflectance of the VNIR subset. Table 7 shows that the results of the OLSR and the CR derived indices were satisfactory for both the MBD and AOM, as long as the absorption feature Z4 were considered, achieving an R^2^ of 0.709 and 0.720, respectively. The suitability of this method decreased rapidly when other absorption features were used. In all cases, AOM yielded more accurate models than MBD for predicting total aboveground grass/clover biomass.

Input data: vid. Table 5; Spectra is the average measurement of the data; R^2^ is the coefficient of determination (cross-validation); RMSE is the Root Mean Square Error (cross-validation); %RMSE is the percentage of Root Mean Square Error (cross-validation) in relation to the average value of the variable.

According to Table 6 and Table 7, the models with the highest R^2^ and lowest RMSE implied the use of data from the region Z4, suggesting the potential of those data to estimate TAGB. Hence, all possible combinations of indices including that region as input data were explored using PLSR. OLSR was not considered in order to avoid colinearity issues. As a result Table 8 shows that the adequate selection of the input data that led to an increase in the accuracy of the model and to a greater simplicity (two latent factors instead of 3 or 4, as in the case of more indices being included), and confirms the PLSR model which considers the MBD index derived from the continuum removed reflectance in the absorption features between 916 and 1,120 nm (Z3) and 1,079 and 1,297 nm (Z4) (R^2^ = 0.800, RMSE = 7.120 g/m^2^) as the most accurate to predict TAGB.

Input data: *vid*. Table 5; F is the number of latent factors; Spectra is the average measurement of the data; R^2^ is the coefficient of determination (cross-validation); RMSE is the Root Mean Square Error (cross-validation); %RMSE is the percentage of Root Mean Square Error (cross-validation) in relation to the average value of the variable.

#### 4.1.2. Results for the Estimation of Green above Ground Biomass

The PLSR and SVM models used to estimate green above ground biomass achieved the smallest RMSE when the largest subset of the spectra (VNIR + SW1 + SW2) was used, reaching values of RMSE smaller than 11% of the average value of the variable (Table 6). As an example, non-transformed data modelled by SVM were able to explain 93.3% of the variance of the data (RMSE = 3.229 g/m^2^, 10.18% of the mean value), while the model developed with PLSR for the same spectral data corrected by the baseline offset transformation showed a similar result (R^2^ = 0.929, RMSE = 3.417 g/m^2^, 10.78% of the mean value). Using the continuum removal transformation did not improve the performance of PLSR when the reflectance values were used as input (Table 6). Nevertheless, the AOM index derived from the continuum removed spectra (Z1, Z3, Z4) provided the most accurate model overall (R^2^ = 0.939, RMSE = 3.172 g/m^2^, 10.00% of the mean value). In addition, the model fitted by OLSR for the AOM index (absorption feature Z4) performed better (R^2^ = 0.914, RMSE = 3.646 g/m^2^, 11.50% of the mean value) than some other PLSR and SVM more complex models (Table 6). Table 7 shows that the results of the OLSR and the CR derived indices were satisfactory for both the MBD and AOM, as long as the absorption feature Z4 were considered, achieving an R^2^ of 0.910 and 0.915, correspondingly. The absorption feature Z3 provided as well a high R^2^ (R^2^ = 0.870 and R^2^ = 0.866). In all cases, AOM yielded more accurate models than MBD for predicting green aboveground grass/clover biomass. The other transformations applied to the PLSR input data did not improve the performance of the algorithm, except for when the VNIR subset and the de-trending using a 3st-order polynomial were considered (R^2^ = 0.901 and RMSE = 4.035 g/m^2^ compared to R^2^ = 0.875 and RMSE = 4.546 g/m^2^, respectively) (Table 7).

**Figure 5.**
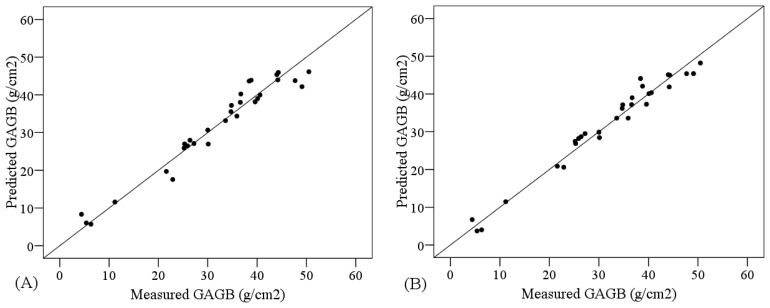
Cross-calibration results for predicting GAGB using (**A**) PLSR based on the continuum-removed AOM index and (**B**) SVM based on VNIR + SWIR1 + SWIR2. One-to-one line is showed.

The comparative analysis of the performance of PLSR models and SVM models showed higher R^2^ and smaller RMSE for the PLSR models only when the non-transformed spectral subsets used were different from VNIR + SW1 + SW2, in which case SVM was the most accurate (Table 6). Figure 5 shows the suitability of the most accurate PLSR and SVM models, according to the cross-validation results. Both models were suitable since the measured and predicted values are distributed along the one-to-one line and close to it.

The PLSR/CRR and OLSR models with the highest R^2^ and lowest RMSE involved the use of data from the region Z4 (Table 6 and Table 7), suggesting the potential of that absorption feature to e stimate GAGB. Thus, all possible combinations of indices including that region as input data were explored using PLSR (combinations of 2, 3, 4 and 5 regions). As for TGAB, OLSR was not considered in order to avoid collinearity issues. The results of this analysis are showed in Table 9, which confirms the PLSR model which considers the AOM index derived from the continuum removed reflectance in the absorption features between 440 and 567 nm (Z1), 916 and 1,120 nm (Z3) and 1,079 and 1,297 nm (Z4) (R^2^ = 0.939, RMSE = 3.172 g/m^2^) as the most accurate to predict GAGB.

Input data: vid. Table 5; F is the number of latent factors; Spectra is the average measurement of the data; R^2^ is the coefficient of determination (cross-validation); RMSE is the Root Mean Square Error (cross-validation); %RMSE is the percentage of Root Mean Square Error (cross-validation) in relation to the average value of the variable.

#### 4.1.3. Results for the Estimation of Percentage of Green above Ground Biomass

The models that produced lower RMSE and higher R^2^ when estimating the percentage of green above ground biomass, were characterised by using the VNIR reflectance as input data (VNIR or the absorption feature Z1) and PLSR and SVM regressions (Table 6). The most accurate model that predicted %GAGB involved PLSR and the continuum removed reflectance values in the absorption feature between 440 and 567 nm (Z1) (R^2^ = 0.762, RMSE = 6.852%). This model produced an error smaller than 10% of the median value of the variable, which made it a highly reliable model regarding this statistic.

**Figure 6.**
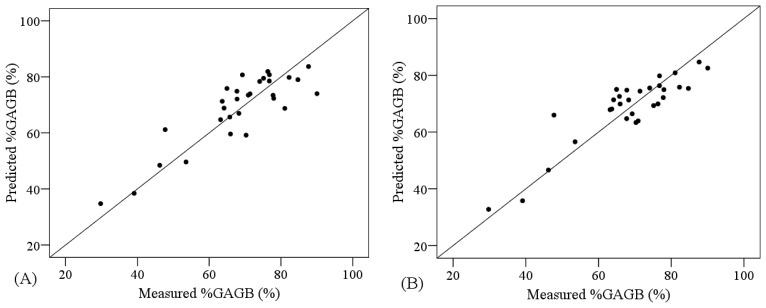
Cross-calibration results for predicting %GAGB using (**A**) PLSR based on the continuum-removed reflectance between 440 and 567 nm (Z1) and (**B**) SVM based on VNIR non-transformed data. One-to-one line is showed.

The combination of PLSR and the VNIR spectra transformed by NGD-3 or RAB produced lower ranges of error for the cross validation analyses (RMSE = 6.919 and 7.500%, respectively) compared to the PLSR applied to non-transformed VNIR data (RMSE = 7.502%) (Table 6). On the other hand, SVM yielded more accurate models than PLSR for non-transformed VNIR data (RMSE = 7.134 and 7.502%, correspondingly). Nonetheless, the comparative analysis of the performance of PLSR models and SVM models showed higher R^2^ and smaller RMSE for the PLSR models when the other regions were considered (VNIR + SW1 and VNIR + SW1 + SW2) (Table 6). Figure 6 shows the degree of suitability of the most accurate PLSR and SVM models, according to the cross-validation results. These models are not as suitable as the ones obtained for the other two AGB variables, since the measured and predicted values are not as homogeneously distributed along the one-to-one line.

The OLSR yielded less accurate models than PLSR and SVM, as it was showed by the fact that the RMSE corresponding to the best OLSR model (AOM in the absorption feature Z5) was 24.08% larger than the RMSE obtained by the best PLSR (Table 6 and Table 7). Since the most accurate models did not involve CR derived indices, the results of exploring all possible combinations of indices including different absorption features using PLSR are not showed.

### 4.2. Discussion

This study has showed the suitability of PLSR and spectral data/indices derived from the CR transformation to estimate the total dry aboveground biomass (TAGB), the green fraction of the dry aboveground biomass (GAGB), and the green fraction of the dry aboveground biomass expressed as a percentage (%GAGB). The results found in our study agree with [28] and [7], which found that PLSR performed better than any other regression methods or narrow banded indices to estimate TAGB (R^2^ = 0.89) and chlorophyll content (R^2^ = 0.85). Moreover [28] found out that PLSR improved the TAGB models by a decrease of 23% in the RMSE in comparison with the models which used NDVI as a predictor. Similar results were achieved by [15], who predicted GAGB more accurately when using CR reflectance and PLSR than with the NDVI index as an independent variable, achieving an R^2^ = 0.83 by applying the former approach. The adequate performance of PLSR to estimate the variables is based on the fact that several biophysical and biochemical variables determine the spectral signature of vegetation canopies [61,62], and therefore indices directly derived from simple band combinations cannot cancel out all the uncertainty introduced by those variables [63], while PLSR is able to do it [7]. It should be noted, however, that the model to estimate %GAGB required more latent factors (7) than TAGB or GAGB (2 and 3 factors, correspondingly), which showed the difficulties faced by PLSR to reduce the level of uncertainty (Table 6).

Transformed data always yielded more accurate models than non-transformed spectral data when the PLSR was applied. However, not always the same transformation improved the results in comparison with not using it. For instance MNX and MSCO were more suitable to model TAGB (RMSE = 7.443 g/m^2^ and RMSE = 7.457 g/m^2^, in comparison with a RMSE = 7.866 g/m^2^ when no transformation was applied), while BLO led to better estimations of GAGB (RMSE = 3.417 g/m^2^*vs.* RMSE = 3.467 g/m^2^) and NGD-3 and RAB did the same for %GAGB (Table 6). [32] achieved comparable results for carbon modelling in soils. The only exception to this result was the CR, which outperformed any other transformation, irrespective of the estimated variable, as showed in [15].

The CR transformation showed that its application on certain regions of the spectra as Z3 (916–1,120 nm) and Z4 (1,079–1,297 nm), boosted the simplification of the TAGB model in comparison to the use of the full non-transformed, as it was epitomised by the decrease in the number of latent factors from 3 to 2 in the PLSR model (Table 6). This result agreed with the ones obtained by [20] for foliar Nitrogen estimation and [15] for grass biomass modelling, and corroborated the hypothesis that an accurate selection of the input data leads to a better performance of the method [15]. The spectral region with largest influence in the estimation of TGAB and GAGB was Z4, which corresponds with the absorption feature between 1079 and 1297 nm, whose bands have been identified as relevant in similar studies by [1,15,23,27,49]. Moreover, %GAGB was best modelled when Z1 (440–567 nm) was the only input data considered, pointing out a relationship between this absorption feature and the percentage of green biomass. It should be noted that the estimation of GAGB also improved when using Z1 data in addition to the Z3 and Z4 regions. The suitability of the Z1 region to model biomass was also acknowledged by [28] and [35], who developed models which achieved R^2^ = 0.89 and R^2^ = 0.61 (respectively) using the Z1 region as input data.

Regarding the use of the two indices derived from the CR transformation (MBD and AOM), the combination of their values in the spectral regions commented previously, yielded the most accurate models for TAGB and GAGB. [1] used these indices to estimate the water content in a field of grass/clover, achieving coefficients of determination (R^2^ = 0,73 and R^2^ = 0,54 for DM y AOM, respectively) comparable to the ones achieved in the present study. The input data used by [1] ranged from 1,115 to 1,270 nm (*i.e.*, similar to the Z4 region), which confirmed the suitability of this part of the spectrum to estimate biomass (TAGB and GAGB).

When no transformations were applied to the reflectance data, SVM outperformed PLSR regarding RMSE when the three variables were estimated. For instance, GAGB was estimated with an RMSE of 3.226 g/m^2^ (10.18%) using SVM, while PLSR led to an RMSE of 3.467 g/m^2^ (10.93%). The better performance of SVM in comparison to PLSR was also noted by [20] and [35] when modelling leaf biochemicals and biomass from spectral data, respectively. They attributed it to the ability of SVM to map non-linear relationships. Nevertheless, other studies show cases where PLSR provided better results than SVM [64]. Those differences might depend on the degree of non-linearity in the relationships, the degree of multicolinearity and noise in the independent variables, and how accurately the SVM parameters can be tuned [20]. Another aspect to consider about SVM is that the accuracy of the model was influenced not only by its parameters, but also by the spectral region considered. As a result, the best region to predict TAGB was VNIR+SWIR1, while for GAGB it was VNIR + SWIR1 + SWIR2, and for %GAGB only the VNIR reflectance was selected as an input. The same regions were chosen by PLSR as predictors for each variable, showing that the optimal predictors depended on the variable of interest and not so much on the algorithm.

## 5. Conclusions

In this paper, it has been demonstrated that the total dry aboveground biomass, as well as the green fraction of the dry aboveground biomass (as an absolute value and as a percentage of the total dry aboveground biomass) can be accurately predicted from spectrometer data by using PLSR and indices derived from the continuum removal transformation of certain regions of the spectra.

The models to estimate the green fraction of the dry aboveground biomass (as an absolute value) yielded smaller errors than the ones predicting the total dry aboveground biomass. Splitting the biomass sample into dry and green fractions allowed the development of more accurate models (for green fraction of the dry aboveground biomass) and it is therefore recommended in case the models need to be recalibrated.

The SVM models provided more accurate estimations of the three variables when no transformations were applied to the reflectance data, which encourages further work to test whether the accuracy of SVM can increase when the input data is previously transformed.

Applying transformations to the data led to more accurate models than non-transformed spectral data when using PLSR. However, unless the continuum removal transformation is chosen, the optimal transformation to apply to the data needs to be identified by taking into account the dependent variable which is being estimated.

Identifying the appropriate absorption features was proven to be crucial in order to improve the performance of PLSR to estimate the total and green aboveground biomass, by using the indices (MBD and AOM) as input data, which are derived from the continuum removed reflectance from those regions. OLSR could be used as a surrogate for the PLSR approach with AOM (1,079–1,297 nm) as the independent variable, although the resulting model would not be as accurate.

## Figures and Tables

**Table 1.** Examples of statistical techniques for estimating vegetation biophysical variables from hyperspectral data.

**Code**	**Technique**	**Examples**
PLSR	Partial least square regression	[3,7,15,19,20,28–35]
SVM	Support vector machine	[20,35–38]
OLSR	Ordinary Least Squares Regression	[1,13,15,18,27,39,40]

**Table 2.** Descriptive statistics of the sample (*n* = 30) (TAGB: total aboveground biomass, GAGB: green portion of the AGB, % GAGB: Percentage of the green faction of the AGB).

**Statistic**	**TAGB(g/m^2^)**	**GAGB (g/m^2^)**	**%GAGB (%)**
Mean	45.05	31.71	68.34
Median	49.10	34.75	69.77
Standard deviation	15.40	12.63	13.57
Maximum	75.60	50.50	90.04
Minimum	9.52	4.40	29.76

**Table 3.** Wavelengths which define the three spectral subsets considered in this research.

**Spectral subset**	**Wavelengths used (nm)**
VNIR	[350–1,000]
VNIR + SWIR 1	[350–1,359], [1,386–1,799]
VNIR +SWIR1 + SWIR2	[350–1,359], [1,386–1,799], [1,931–2,399]

**Table 4.** Pre-processing transformations compared in this study.

**Code**	**Pre-Processing Transformation**	**Examples**
BLO	Baseline offset	[47]

CR	Continuum Removal	[1,13,15,19,23,27,48,49]

DE-TREN1	De-trending using a 1st-order polynomial	[19]
DE-TREN2	De-trending using a 2st-order polynomial	
DE-TREN3	De-trending using a 3st-order polynomial	

MSCA	Multiplicative Scatter Correction Common amplification f(X = X/b)	[19,31,33,34,47,50]
MSCF	Multiplicative Scatter Correction Full MSC f(X) = (X − a)/b	
MSCO	Multiplicative Scatter Correction Common off set f(X) = X − a	

NAR	Normalise by the area	[32]
NMX	Normalise by the maximum value	
NME	Normalise by the mean	
NRA	Normalise by the range	
NUV	Normalise by the unit vector	

NGD-3	Norris gap derivative 1st derivative-gap size = 3	[32]
NGD-5	Norris gap derivative 1st derivative-gap size = 5	
NGD-7	Norris gap derivative 1st derivative-gap size = 7	
NGD-9	Norris gap derivative 1st derivative-gap size = 9	

RAB	Reflectance to absorbance	[32]

SNV	Standard normal variate transformation	[19,31–33,47,51,52]

**Table 5.** Continuum removal zones considered in this study.

**Zone**	**Continuum Range (Nm)**	**Electromagnetic Region**
Z1	[440–567]	VNIR
Z2	[554–762]	VNIR
Z3	[916–1,120]	VNIR+SWIR1
Z4	[1,079–1,297]	SWIR1
Z5	[1,265–1,676]	SWIR1

**Table 6.** Performance of PLSR, SVM and OLSR and spectral transformations for predicting total (TAGB), green (GAGB) and percentage of green (%GAGB) grass/clover biomass.

**Var.**	**Regression Model/Transformation**	**Input Data**	**Spectra**	**F/C**	**R^2^**	**RMSE (g/m^2^)**	**%RMSE**
TAGB	PLSR/MBD	Z3-Z4 (MBD)	Mean	2	0.800	7.120	15.81
	PLSR/CRR	Z4	Mean	5	0.799	7.136	15.84
	PLSR/NMX	VNIR	Mean	6	0.782	7.443	16.52
	PLSR/MSCO	VNIR + SWIR1	Mean	3	0.781	7.457	16.55
	PLSR/MSCO	VNIR + SWIR1+SWIR2	Mean	3	0.770	7.640	16.96
	PLSR/none	VNIR + SWIR1	Mean	3	0.756	7.866	17.46
	SVM/none	VNIR + SWIR1	Mean	0.04	0.751	7.684	17.06
	PLSR/none	VNIR + SWIR1+SWIR2	Mean	3	0.751	7.950	17.65
	SVM/none	VNIR + SWIR1+SWIR2	Mean	0.03	0.745	7.780	17.27
	OLSR/AOM	Z4 (AOM)	Mean	1	0.720	8.150	18.09
	PLSR/none	VNIR	Median	3	0.689	8.888	19.73
	SVM/none	VNIR	Median	0.11	0.683	8.690	19.29

GAGB	PLSR/AOM	Z1-Z3-Z4 (AOM)	Mean	3	0.939	3.172	10.00
	SVM/none	VNIR + SWIR1 + SWIR2	Mean	0.1	0.933	3.229	10.18
	PLSR/BLO	VNIR + SWIR1 + SWIR2	Mean	6	0.929	3.417	10.78
	PLSR/none	VNIR + SWIR1 + SWIR2	Mean	6	0.927	3.467	10.93
	PLSR/CRR	Z4	Median	1	0.921	3.622	11.42
	OLSR/AOM	Z4 (AOM)	Mean	1	0.914	3.646	11.50
	PLSR/none	VNIR + SWIR1	Mean	5	0.913	3.789	11.95
	SVM/none	VNIR + SWIR1	Mean	0.14	0.909	3.759	11.85
	PLSR/DE-TREN3	VNIR	Mean	4	0.901	4.035	12.72
	PLSR/MSCO	VNIR + SWIR1	Mean	3	0.901	4.036	12.73
	PLSR/none	VNIR	Median	6	0.875	4.546	14.34
	SVM/none	VNIR	Median	0.16	0.846	4.895	15.44

%GAGB	PLSR/CRR	Z1	Median	7	0.762	6.852	9.82
	PLSR/NGD-3	VNIR	Mean	4	0.757	6.919	10.12
	SVM/none	VNIR	Mean	0.07	0.724	7.134	10.44
	PLSR/RAB	VNIR + SWIR1	Mean	5	0.715	7.500	10.97
	PLSR/none	VNIR	Median	3	0.714	7.502	10.75
	PLSR/NAR	VNIR + SWIR1 + SWIR2	Mean	3	0.705	7.628	11.16
	PLSR/AOM	Z2-Z3-Z5 (AOM)	Median	3	0.684	7.897	11.32
	PLSR/none	VNIR + SWIR1 + SWIR2	Median	4	0.682	7.913	11.34
	PLSR/none	VNIR + SWIR1	Median	3	0.678	7.947	11.39
	SVM/none	VNIR + SWIR1	Mean	0.02	0.650	8.047	11.53
	SVM/none	VNIR + SWIR1 + SWIR2	Median	0.02	0.655	7.991	11.69
	OLSR/MBD	Z5	Median	1	0.608	8.502	12.19

**Table 7.** Performance of Ordinary Least Squares Regression (OLSR), for predicting total (TAGB), green (GAGB) and percentage of green (%GAGB) grass/clover biomass using indices derived from the continuum removed spectra. In bold: most accurate models.

	**Maximum Band Depth (MBD)**				**Area Over the Minimum (AOM)**			
	
	**Input**	**Spectra**	**R^2^**	**RMSE (g/m^2^)**	**Input**	**Spectra**	**R^2^**	**RMSE (g/m^2^)**
TAGB	Z1	Median	0.582	9.950	Z1	Mean	0.594	9.810
	Z2	Mean	0.537	10.476	Z2	Median	0.577	10.008
	Z3	Mean	0.650	9.110	Z3	Mean	0.641	9.226
	**Z4**	**Mean**	**0.709**	**8.301**	**Z4**	**Mean**	**0.720**	**8.150**
	Z5	Mean	0.599	9.748	Z5	Mean	0.642	9.216

GAGB	Z1	Median	0.728	6.483	Z1	Mean	0.722	6.546
	Z2	Median	0.669	7.146	Z2	Median	0.719	6.587
	Z3	Mean	0.870	4.470	Z3	Mean	0.866	4.550
	**Z4**	**Median**	**0.910**	**3.720**	**Z4**	**Median**	**0.915**	**3.615**
	Z5	Mean	0.743	6.293	Z5	Median	0.797	5.593

%GAGB	Z1	Median	0.567	8.931	Z1	Median	0.554	9.064
	Z2	Median	0.603	8.551	Z2	Median	0.591	8.674
	Z3	Median	0.557	9.034	Z3	Mean	0.552	9.080
	Z4	Median	0.524	9.359	Z4	Mean	0.523	9.370
	**Z5**	**Median**	**0.608**	**8.502**	**Z5**	**Median**	**0.594**	**8.648**

**Table 8.** Performance of PLSR for predicting total (TAGB) grass/clover biomass using indices which consider the absorption feature Z4 derived from the continuum removed spectra. In bold: most accurate models.

**Input data**	**MBD**				**AOM**			
	
	**F**	**Spectra**	**R^2^**	**RMSE (g/m^2^)**	**F**	**Spectra**	**R^2^**	**RMSE (g/m^2^)**
Z1-Z4	2	Median	0.709	8.595	2	Median	0.708	8.612
Z2-Z4	2	Median	0.724	8.369	2	Median	0.721	8.406
**Z3-Z4**	**2**	**Median**	**0.800**	**7.120**	**2**	**Median**	**0.719**	**8.436**
Z4-Z5	2	Median	0.725	8.347	2	Median	0.723	8.386

Z1-Z2-Z4	2	Median	0.683	8.971	2	Median	0.679	9.023
Z1-Z3-Z4	3	Median	0.786	7.375	3	Median	0.727	8.317
Z1-Z4-Z5	2	Median	0.685	8.934	2	Median	0.692	8.838
**Z2-Z3-Z4**	**3**	**Median**	**0.792**	**7.270**	**2**	**Mean**	**0.736**	**8.178**
Z2-Z4-Z5	2	Mean	0.710	8.579	2	Mean	0.705	8.646
Z3-Z4-Z5	3	Median	0.794	7.237	3	Median	0.730	8.274

Z1-Z2-Z3-Z4	4	Median	0.772	7.599	3	Median	0.739	8.144
Z1-Z2-Z4-Z5	2	Mean	0.679	9.025	2	Median	0.672	9.121
Z1-Z3-Z4-Z5	4	Median	0.772	7.611	3	Median	0.716	8.489
Z2-Z3-Z4-Z5	4	Median	0.780	7.473	2	Median	0.725	8.347

Z1-Z2-Z3-Z4-Z5	5	Median	0.758	7.832	2	Mean	0.724	8.360

**Table 9.** Performance of PLSR for predicting green (GAGB) grass/clover biomass using indices which consider the absorption feature Z4 derived from the continuum removed spectra. In bold: most accurate models.

**Input Data**	**MBD**				**AOM**			
	
	**F**	**Spectra**	**R^2^**	**RMSE (g/m^2^)**	**F**	**Spectra**	**R^2^**	**RMSE (g/m^2^)**
Z1-Z4	2	Mean	0.914	3.762	2	Mean	0.924	3.539
Z2-Z4	2	Median	0.913	3.783	2	Mean	0.918	3.675
**Z3-Z4**	**2**	**Mean**	**0.919**	**3.661**	**2**	**Mean**	**0.929**	**3.433**
Z4-Z5	2	Median	0.913	3.786	2	Median	0.921	3.607

Z1-Z2-Z4	3	Median	0.915	3.745	3	Median	0.925	3.512
**Z1-Z3-Z4**	**3**	**Median**	**0.923**	**3.566**	**3**	**Mean**	**0.939**	**3.172**
Z1-Z4-Z5	2	Mean	0.918	3.689	3	Mean	0.922	3.599
Z2-Z3-Z4	3	Mean	0.915	3.738	3	Mean	0.929	3.432
Z2-Z4-Z5	2	Mean	0.900	4.060	3	Median	0.921	3.607
Z3-Z4-Z5	3	Mean	0.915	3.755	3	Mean	0.931	3.377

**Z1-Z2-Z3-Z4**	**3**	**Median**	**0.922**	**3.588**	**4**	**Mean**	**0.939**	**3.173**
Z1-Z2-Z4-Z5	3	Mean	0.913	3.782	4	Median	0.921	3.618
Z1-Z3-Z4-Z5	3	Median	0.917	3.703	4	Mean	0.936	3.249
Z2-Z3-Z4-Z5	2	Mean	0.898	4.098	4	Mean	0.929	3.427

Z1-Z2-Z3-Z4-Z5	3	Median	0.917	3.702	5	Mean	0.931	3.367
